# Case of Separation of the Stomach from the Œsophagus

**Published:** 1849-05

**Authors:** 


*Case of Separation of the Stomach from the Œsophagus.*—By Thomas M. Flint.

Prof. Dunglison.

*Dear Sir* :—By your request I furnish you the particulars of the case in which softening of the stomach was found to have occurred. The attending physician, who is a respectable graduate of this school, has given me the following statement of facts: "The patient was a male child; aged seven years; sick about three weeks; symptoms of worms were prominent — one was passed; cerebral symptoms followed, which terminated in death. Coma and unconsciousness were prominent symptoms for ten days previous to death. When roused from this state he would eat a small quantity of gruel. He was treated for worms and cerebral symptoms."

On the 4th inst., thirty-six hours after death, I opened the body in the presence of the attending physician and a member of this class. We carefully examined the intestines, beginning at the rectum and tracing the tube up to the connection of the duodenum with the stomach, without meeting with a worm of any kind; but noticed marked inflamation of the small intestines. We next directed our attention to the stomach itself, which, to our surprise, was found to be severed from its connection with the œsophagus, and its contents, a dark-brownish mucilaginous-like fluid, poured out into the cavity of the abdomen to the left of the spinal column. We were not prepared to meet with a lesion of this character, and could account for it only by the action of the gastric acids producing *ramollissement* of this organ after death. In this opinion we were confirmed by the appearance of the liver; for beside evident marks of acute inflammation, the inferior edge of the left lobe, which had been in contact with the gastric fluid, was corroded and corrugated.

That you may have the opportunity of examining the case, I herewith deliver to you the stomach and liver taken from the patient at tne post-mortem.—*Med. Examiner.*

